# Gut Bacteria *Erysipelatoclostridium* and Its Related Metabolite Ptilosteroid A Could Predict Radiation-Induced Intestinal Injury

**DOI:** 10.3389/fpubh.2022.862598

**Published:** 2022-03-28

**Authors:** Shang Cai, Yongqiang Yang, Yuehong Kong, Qi Guo, Yingying Xu, Pengfei Xing, Yanze Sun, Jianjun Qian, Ruizhe Xu, Liwei Xie, Yijia Hu, Min Wang, Ming Li, Ye Tian, Weidong Mao

**Affiliations:** ^1^Department of Radiotherapy and Oncology, The Second Affiliated Hospital of Soochow University, Suzhou, China; ^2^Institute of Radiotherapy and Oncology, Soochow University, Suzhou, China; ^3^Suzhou Key Laboratory for Radiation Oncology, Suzhou, China; ^4^Department of Clinical Laboratory, The Second Affiliated Hospital of Soochow University, Suzhou, China; ^5^State Key Laboratory of Radiation Medicine and Protection, School of Radiation Medicine and Protection, Medical College of Soochow University, Collaborative Innovation Center of Radiation Medicine of Jiangsu Higher Education Institutions, Soochow University, Suzhou, China

**Keywords:** radiation-induced intestinal injury, biomarker, gut bacteria, gut bacteria related metabolite, radiotherapy

## Abstract

It is difficult to study the intestinal damage induced by space radiation to astronauts directly, and few prediction models exist. However, we can simulate it in patients with pelvic tumor radiotherapy (RT). Radiation-induced intestinal injury (RIII) is common in cancer patients who receieved pelvic and abdominal RT. We dynamically analyzed gut microbiota and metabolites alterations in 17 cervical and endometrial cancer patients after pelvic RT. In patients who later developed grade 2 RIII, dysbiosis of gut microbiota and metabolites were observed. Univariate analysis showed that *Erysipelatoclostridium* and ptilosteroid A were related to the occurrence of grade 2 RIII. Notably, a strong positive correlation between gut bacteria *Erysipelatoclostridium* relative abundance and gut metabolite ptilosteroid A expression was found. Furthermore, combinations of *Erysipelatoclostridium* and ptilosteroid A could provide good diagnostic markers for grade 2 RIII. In conclusion, gut bacteria *Erysipelatoclostridium* and its related metabolite ptilosteroid A may collaboratively predict RIII, and could be diagnostic biomarkers for RIII and space radiation injury.

## Introduction

Life science in space research is committed to understanding the basic laws of life activities in the space environment, and exploring the natural phenomena and laws of life in the universe. However, the extreme environments such as space radiation can also cause serious damage to astronauts. The space radiation environment is an extreme environment that has not been encountered in the evolution of Earth's organisms, and because of the space environment cannot be simulated on the ground, there are still many problems and key technologies to be overcome in the research in this field. In this paper, we used the intestinal damage caused by pelvic tumor radiotherapy as a model to evaluate the impact of radiation on human intestinal damage and intestinal microbes, hoping to provide a research basis for the damage mechanism and protection strategies of space radiation.

Radiotherapy (RT) is an essential modality in multi-disciplinary treatment for pelvic and abdominal malignancies (1). However, the risk of normal tissue toxicities restricts the RT application. Among them, radiation-induced intestinal injury (RIII) is one of the main problems (2–4). Since RIII lacks efficient clinical treatment method (5, 6), development of bio-markers for prediction of RIII risk are in urgent need.

Intestinal microbiota influences a range of physiological and pathological processes *via* generating bioactive compound: microbiota–derived metabolites. Therefore, gut microbiota and their derived metabolites not only reflect local intestinal ecosystem, but also provide information about host homeostasis, and may serve as potential bio-markers of RIII and space radiation injury (7–9).

To the best of our knowledge, this study is one of the first attempts to establish the intestinal microbiota—and metabolite—based prediction model of RIII to space radiation damage. In this prospective, observational clinical study, we dynamically explored gut microbiota and their derived metabolite alterations in response to pelvic RT. Base on that, we further established a 2-variable model that contains gut bacteria and its related metabolite for the prediction of RIII. Our results could provide an easily obtainable clinical biomarkers to aid in early diagnoses of RIII and space radiation damage.

## Materials and Methods

### Patient Recruitment and Characteristics

This study was approved by the ethic committee of The Second Affiliated Hospital of Soochow University (JD-LK-2017-013-02), and was conducted according to the principles of the Declaration of Helsinki. Inclusion criteria were: (1) newly diagnosed and pathologically confirmed stage I to III cervical or endometrial cancer; (2) receive pelvic or abdominal RT for the first time; (3) KPS ≥ 70. RT treatments were conducted according to the RTOG or ESTRO guidelines. Stool samples were collected before, 20–30 and 45–50 Gy after RT, respectively. All samples were aliquoted and stored at −80°C until further use.

### Follow-Up and Assessment of RIII

Patients were examined at least once before RT and weekly during RT. Then, patients were examined 1 week, 1 month, and 3 months after RT completion, and every 3 months thereafter. RIII was diagnosed and scored according to the RTOG acute radiation morbidity scoring criteria.

### DNA Extraction and Sequencing of 16S rRNA Gene

Briefly, bacterial genomic DNA was extracted by using the QIAamp Fast DNA Mini Kit (Qiagen, Hilden, Germany), and all the operations were conducted following the manufacturer's instructions. Then, the 16S rRNA V3–V4 variable regions were amplified with universal primers (343F and 798R) by PCR. Finally, sequencing was conducted by using the Illumina HiSeq2500 platform.

### LC-MS Analysis

LC-MS analysis was performed using ACQUITY UPLC I-Class system (Waters Corporation, Milford, USA) coupled with VION IMS QTOF Mass spectrometer (Waters Corporation, Milford, USA) system with an ACQUITY UPLC BEH C18 column (1.7 μm, 2.1 X 100 mm). Mobile phases A and B were using water and acetonitrile/methanol 2/3(v/v), both containing 0.1% formic acid, respectively. The linear gradient was: 0 min, 1% B; 1 min, 30% B; 2.5 min, 60% B; 6.5 min, 90% B; 8.5 min, 100% B; 10.7 min, 100% B; 10.8 min, 1% B, and 13 min, 1%B. The column temperature was 45°C and the flow rate was 0.4 mL/min.

### Statistical Analysis and Bioinformatics

16S rRNA sequencing raw data were firstly preprocessed *via* Trimmomatic software, then converted to generate operational taxonomic units (OTUs) by using Vsearch software with 97% similarity as cutoff value. Next, α-diversity was assessed by Chao1 and Shannon indexes. β-diversity was assessed by PCoA, NMDS, Adonis and Anosim analysis. Further, the differential expressed microbiota were analyzed by ANOVA, Kruskal Wallis, *T*-test, Wilcoxon and LEfSe test.

LC-MS raw data were analyzed by QI software (Waters Corporation, Milford, USA). Partial least squares discrimination analysis (PLS-DA) and orthogonal PLS-DA (OPLS-DA) were performed to visualize the metabolic alterations between different groups. The downstream potential enriched metabolic pathways were acquired by performing KEGG pathway analysis.

Student's *t*-test or Chi-square test were carried out to compare variables between different groups. Univariate logistic regression analysis was used to identify potential biomarkers, and receiver operator characteristic (ROC) curves were constructed. Spearman's correlation test was performed to detect the correlation between expression levels of gut *Erysipelatoclostridium* and ptilosteroid A. Statistical analysis was performed using SPSS version 22 software (IBM Corp., Armonk, NY), and *p*-value < 0.05 was considered significant.

## Results

### Study Population and Characteristics

Totally, 17 patients (16 cervical cancer and one endometrial cancer) were recruited, and their characteristics are shown in Table 1. Among them, 11 (65%) developed grade 2 RIII, 2 (12%) developed grade 1 RIII, and 4 (23%) did not develop any grade of RIII. Since the symptoms of grade 1 RIII is mild and with no need for specific treatment, patients were divided into grade 2 RIII group and grade 0 or 1 RIII group for further analysis. As we can see, no differences in age, RT type, whether acceptance of concurrent chemotherapy or not, tumor stage and dosimetric parameters of gut were found between the two groups (Tables 2, 3).

**Table 1:** Clinical features of patients.

**Characteristics**	**Values**
**Age (years)**	52 (range 35–77)
**Karnofsky performance score**	
≥70	17 (100%)
<70	0 (0%)
**Radiotherapy**	
Radical	6 (35%)
Adjuvant	11 (65%)
**Concurrent chemotherapy**	
Yes	10 (59%)
None	7 (41%)
**Stage (I/II/III)**	
I	2 (12%)
II	6 (35%)
III	9 (53%)
**Radiation enteritis**	
0–1	3 (35%)
>2	11 (65%)

**Table 2:** Clinical features of grade 2 and grade 0 or 1 RIII patients.

**Characteristics**	**2 RIII**	**0–1 RIII**	***P*-values**
	**11**	**6**	
**Age (years)**			
≥50	4	2	0.661
<50	7	4	
**Radiotherapy**			
Radical	3	3	0.339
Adjuvant	8	3	
**Concurrent chemotherapy**			
Yes	8	2	0.682
None	3	4	
**Stage (I/II/III)**			
I	1	1	0.898
II	4	2	
III	6	3	

**Table 3:** Dosimetric parameters of grade 2 and grade 0 or 1 RIII patients.

**Characteristics**	**2 RIII**	**0–1 RIII**	***P*-values**
V50 (%, small intestine)	7.67 ± 2.98	6.73 ± 2.45	0.83
V40 (%, small intestine)	28.76 ± 3.83	25.55 ± 4.47	0.6
V30 (%, small intestine)	44.51 ± 4.53	42.68 ± 6.34	0.81
D_max_ (cGy, small intestine)	4,639 ± 581	5,328 ± 200	0.37
D_mean_ (cGy, small intestine)	2,642 ± 187	3,180 ± 259	0.11
V50 (%, rectum)	23.93 ± 10.32	27.05 ± 9.93	0.84
V40 (%, rectum)	88.42 ± 2.89	78.17 ± 7.65	0.27
V30 (%, rectum)	97.35 ± 1.76	96.12 ± 1.87	0.65
D_max_ (cGy, rectum)	5,134 ± 99	5,178 ± 114	0.78
D_mean_ (cGy, rectum)	4,379 ± 206	4,544 ± 180	0.58

### Differences in Gut Microbiome Between Grade 2 and Grade 0 or 1 RIII Patients

**Figure 1:**
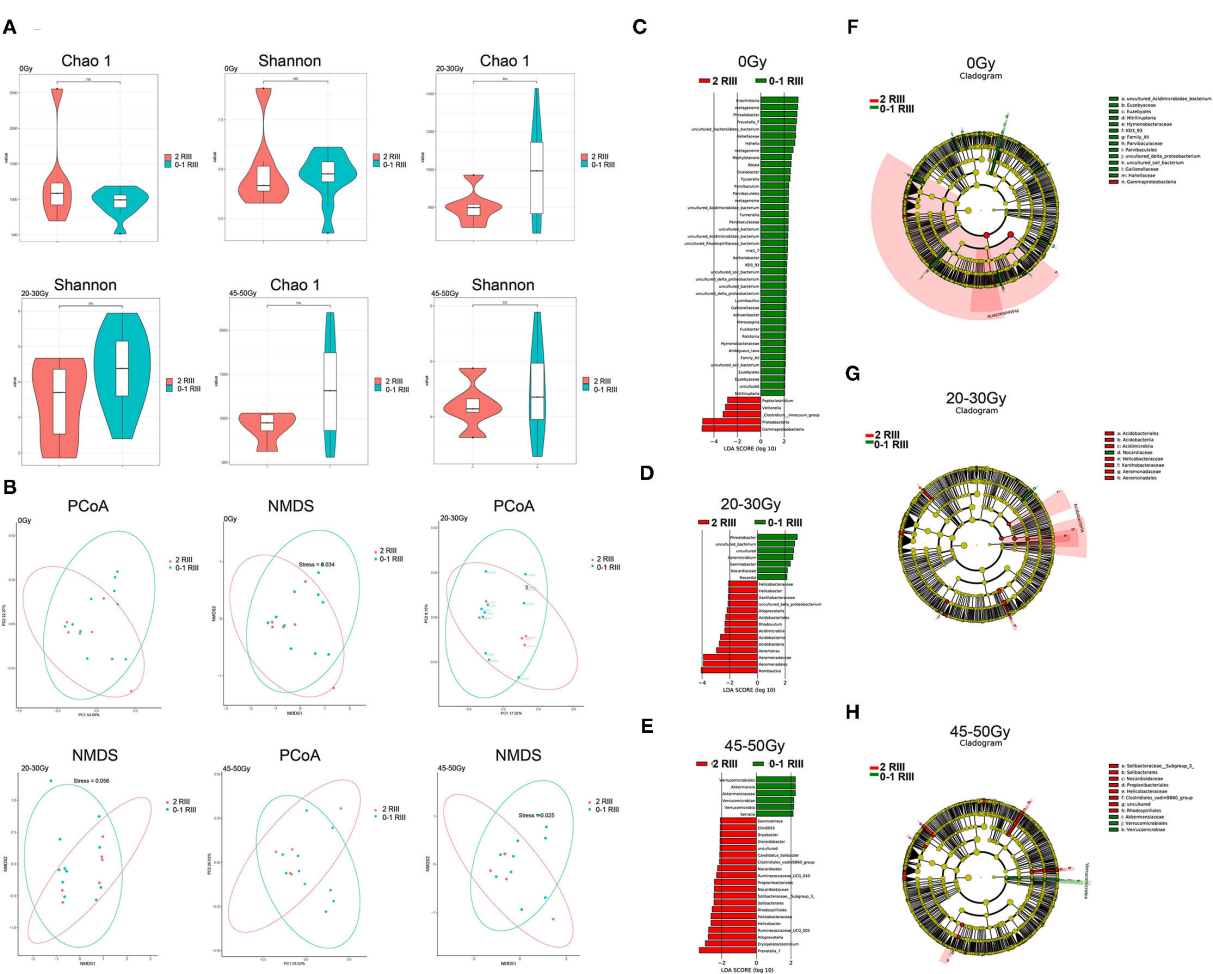
Alterations of gut microbiome between grade 2 and grade 0 or 1 RIII patients at all the three timepoints. **(A)** Box plot of the Chao1 index and Shannon index. **(B)** PCoA and NMDS analysis. **(C–E)** LEfSe analysis based on OTU abundance. **(F–H)** Taxonomic cladograms with LDA score ≥2.

We firstly compared α-diversity and β-diversity between the two groups, respectively. For the α-diversity analysis, we found no significant differences (Figure 1A). As for the β-diversity, partial distinction was found (Figure 1B), indicating that the heterogeneousness rather than the complexity of gut microbiota community was partly distinct between grade 2 and grade 0 or 1 RIII patients. Next, we try to identify the RIII-related bacterial taxa by LEfSe. As we can see, at genus level, 47, 22, and 26 discriminative features with LDA score ≥2 were identified before or after 20–30 and 45–50 Gy, respectively (Figures 1C–E). Further, the relationship between taxa at different taxonomic levels of these discriminative features were represented (Figures 1F–H).

### Gut Metabonomics in Grade 2 RIII Patients Significantly Differed From That in Grade 0 or 1 RIII Patients

**Figure 2:**
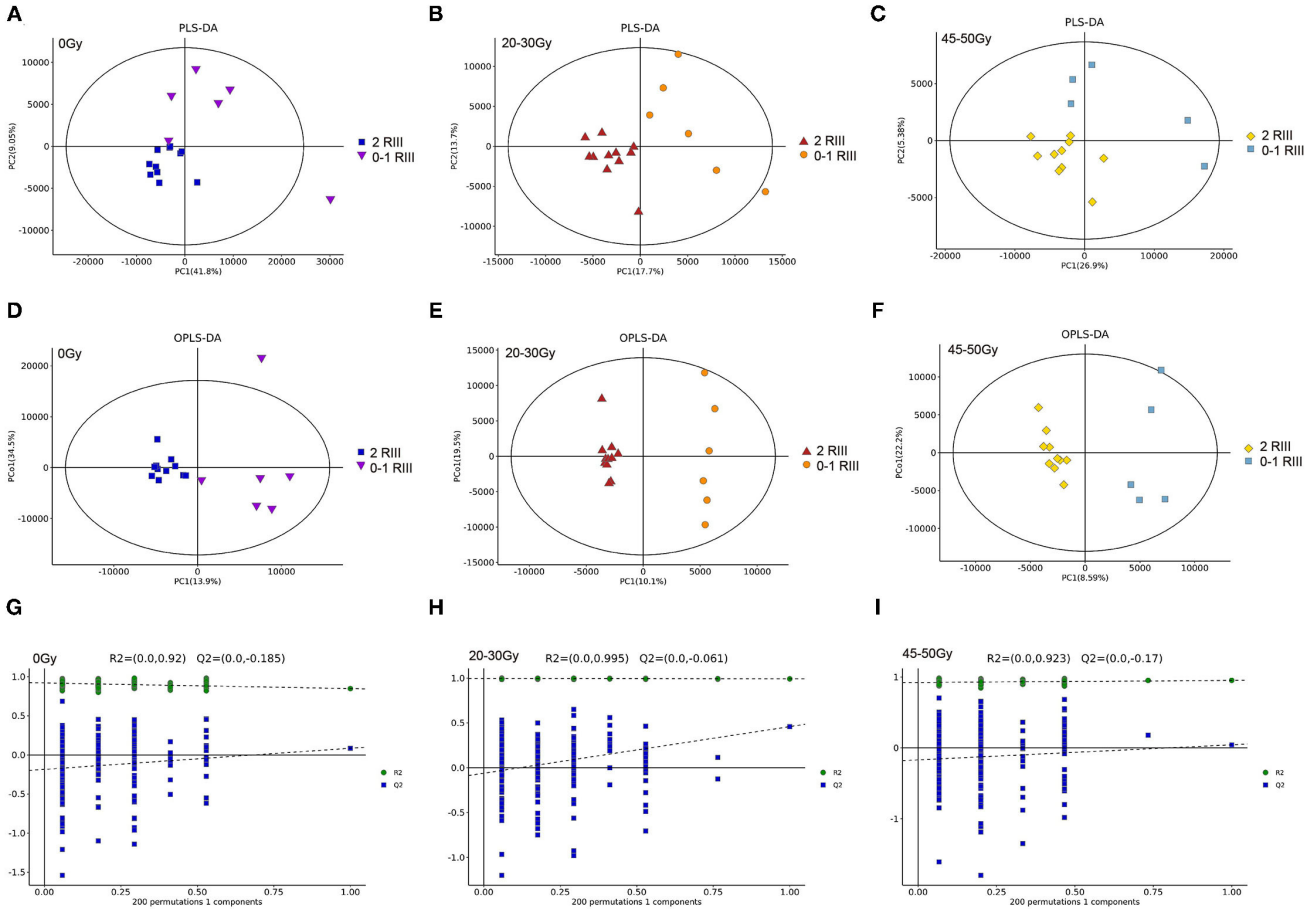
Alterations of gut metabolites between grade 2 and grade 0 or 1 RIII patients at all the three timepoints. **(A–F)** PLS-DA and OPLS-DA score plots. **(G–I)** OPLS-DA score plots.

For all the three timepoints, both PLS-DA and OPLS-DA analysis identified two distinct metabolite profiles between the two groups (Figures 2A–F), indicating that gut metabonomics differed markedly between grade 2 RIII and grade 0 or 1 RIII patients. Further, permutation test was conducted, and the R2 and Q2 values indicated the suitability for subsequent optimization analyses (Figures 2G–I).

### Gut Metabonomics in Grade 2 RIII Patients Significantly Differed From That in Grade 0 or 1 RIII Patients

**Figure 3:**
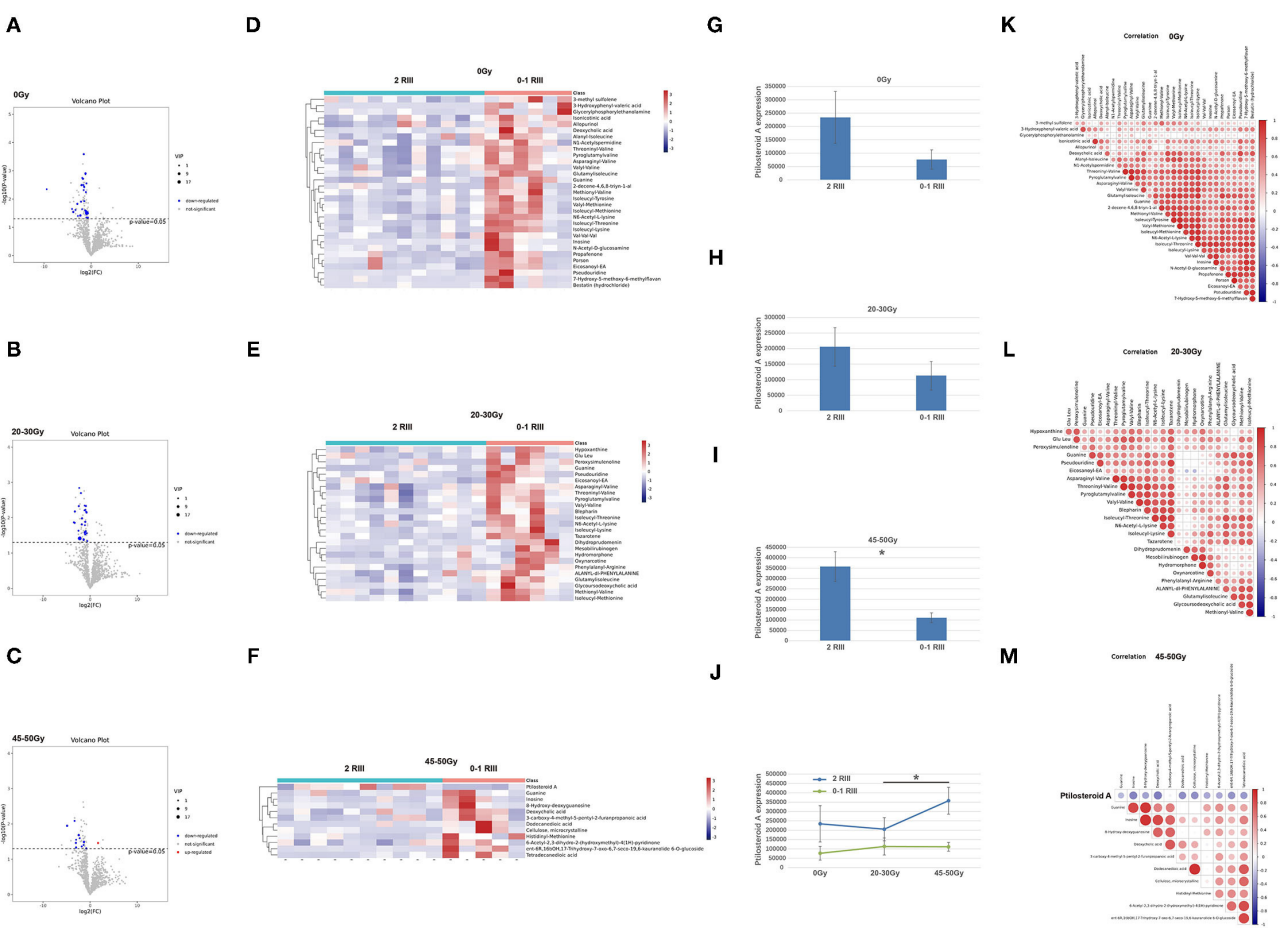
Gut metabonomics in grade 2 RIII patients significantly differed from that in grade 0 or 1 RIII patients at all the three timepoints. **(A–C)** Volcano plot of the significantly differential metabolites. **(D–F)** Heat maps of the significantly differential metabolites. **(G–J)** Expression level of ptilosteroid A of the two groups. **(K–M)** correlations of the differentially expressed metabolites. The symbol * means the *P* value was smaller than 0.05.

As shown in the volcano plot (Figures 3A–C), there were 31, 25, and 12 differential metabolites between grade 2 RIII group and grade 0 or 1 RIII group before or after 20–30 and 45–50 Gy, respectively. Heatmap was also presented (Figures 3D–F). Among them, ptilosteroid A was the only one that enriched in the grade 2 RIII group (Figure 3F). Then we measured gut ptilosteroid A levels between the two groups. We found that before and after 20–30 Gy of RT, the ptilosteroid A levels in the grade 2 RIII group were higher than that of grade 0 or 1 RIII group, but was not significant (Figures 3G,H). However, the difference of ptilosteroid A level between the two groups became significant after 45–50 Gy (Figure 3I). During the course of RT, for the patients with grade 2 RIII their gut ptilosteroid A level kept increasing and reached the highest level (1.7-fold) after 45–50 Gy, but not for that with grade 0 or 1 RIII patients (Figure 3J). Therefore, ptilosteroid A was selected as a potential biomarker for RIII. Next, for these differentially expressed metabolites identified above, their correlations were analyzed by Pearson linear correlation test, and was presented (Figures 3K–M).

### Identification of Significantly Different Pathways Associated With RIII

**Figure 4:**
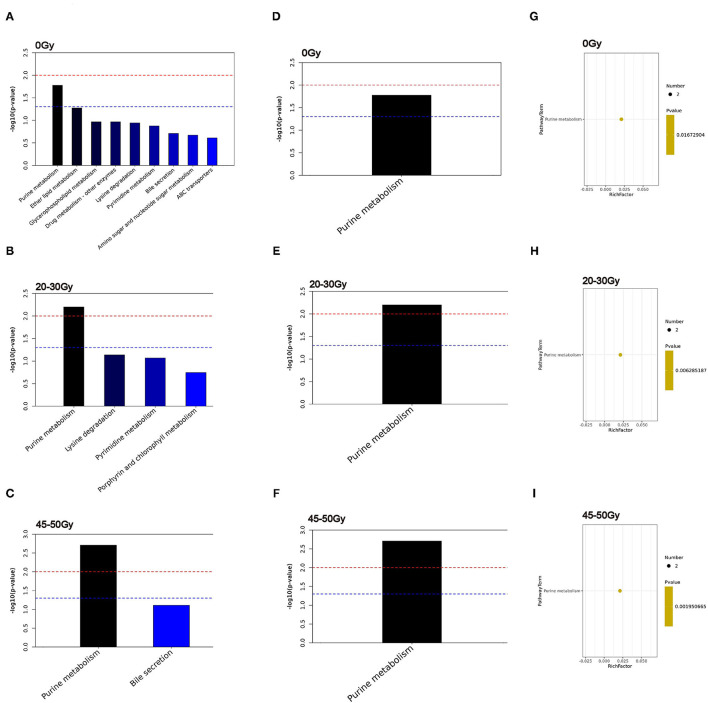
Identification of significantly different pathways associated with RIII. **(A–C)** The top 20 enriched pathways. **(D–F)** Heatmap of enriched metabolic pathway. **(G–I)** bubble plots of enriched metabolic pathway.

Additionally, KEGG pathway analysis was conducted (Figures 4A–C), among them the purine metabolism was the only one enriched pathway associated with the occurrence of RIII (Figures 4D–I).

### Predictive Value of Clinical Characteristics, Gut Microbiome, and Metabolites for Grade 2 RIII

**Figure 5:**
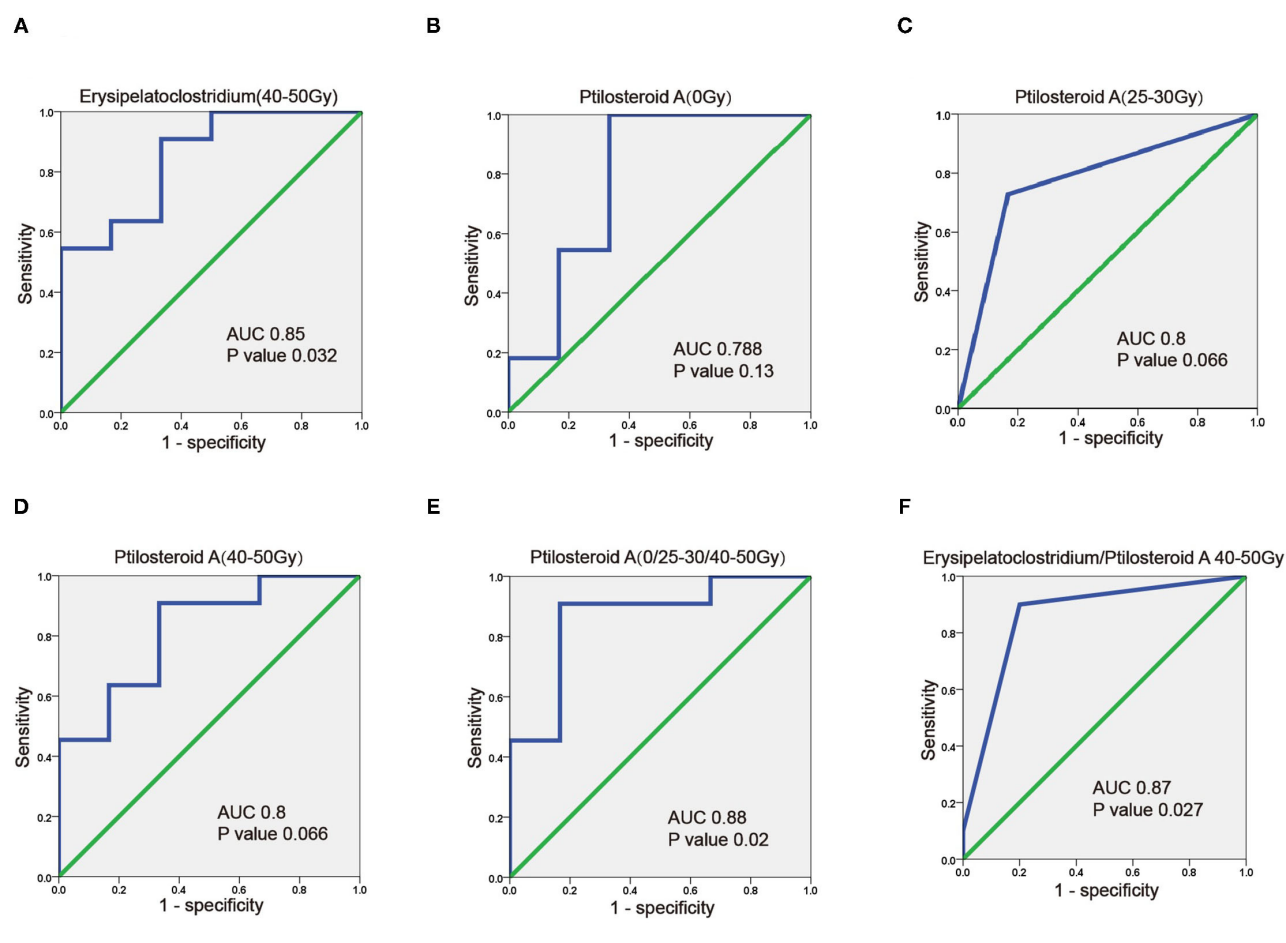
Predictive value of gut microbiome and metabolites for predicting RIII. **(A)** ROC plot for *Erysipelatoclostridium* after 45–50 Gy. **(B–D)** ROC plot for ptilosteroid A at 0 Gy, after 20–30 and 45–50 Gy. **(E)** The ROC analysis of the combination of ptilosteroid A at all the three timepoints. **(F)** The ROC analysis of the combination of *Erysipelatoclostridium* and ptilosteroid A.

In order to seek out potential bio-markers for RIII, clinical factors gut microbiome and metabolites identified above, were tested by univariate analysis. However, none clinical factors were related to the occurrence of grade 2 RIII (Table 4). For the gut microbiome, the relative abundance of *Erysipelatoclostridium* after 45–50 Gy was related to the occurrence of grade 2 RIII. As for the metabolites, the expression level of ptilosteroid A at all the three timepoints were related to the occurrence of grade 2 RIII. Therefore, gut microbial *Erysipelatoclostridium* and metabolite ptilosteroid A were chosen as potential bio-markers for further study. Next, the predictive ability of gut microbial *Erysipelatoclostridium* and metabolite ptilosteroid A for RIII was assessed by ROC analysis. Single variable model containing gut microbial *Erysipelatoclostridium* at 45–50 Gy exhibited AUC value of 0.85 (Figure 5A). Meanwhile, single variable model containing gut metabolite ptilosteroid A exhibited ATC value of 0.788 (0 Gy), 0.8 (after 20–30 Gy), and 0.8 (after 45–50 Gy), respectively (Figures 5B–D). Interestingly, better predictive ability can be obtained by combining ptilosteroid A of all the three timepoints or ptilosteroid A with *Erysipelatoclostridium* (Figures 5E,F).

**Table 4:** Univariate analysis of predictive factors associated with grade 2 RIII.

**Factor**	**Univariate analysis**		
	**OR**	**95% CI**	***P*-values**
Age	0.877	0.768–1	0.5
RT	0.375	0.047–2.998	0.355
CT	5.333	0.618–45.991	0.128
PCT	1	0.112–8.947	0.206
CRT	1.875	0.15–23.396	0.625
V50 (small intestine)	1.011	0.883–1.156	0.878
V40 (small intestine)	1.009	0.919–1.107	0.857
V30 (small intestine)	0.999	0.926–1.079	0.987
D_max_ (small intestine)	0.375	0.996–1.002	0.456
D_mean_ (small intestine)	0.998	0.995–1	0.106
V50 (rectum)	0.994	0.957–1.032	0.752
V40 (rectum)	1.054	0.964–1.152	0.245
V30 (rectum)	1.066	0.859–1.325	0.561
D_max_ (rectum)	0.999	0.995–1.003	0.734
D_mean_ (rectum)	0.999	0.997–1.002	0.601
*Erysipelatoclostridium* (0 Gy)	10	0.739–135.27	0.083
*Erysipelatoclostridium* (25–30 Gy)	2.819	0.229–34.645	0.418
*Erysipelatoclostridium* (40–50 Gy)	36	1.772–331.02	0.020*
Ptilosteroid A (0 Gy)	20	1.391–287.6	0.028*
Ptilosteroid A (25–30 Gy)	13.333	1.069–166.374	0.044*
Ptilosteroid A (40–50 Gy)	16	1.093–234.248	0.043*

The bold values indicate the P value was smaller than 0.05. The * and red highlights indicates these factors were related to the grade 2 RIII.

### Gut Microbial *Erysipelatoclostridium* Positively Correlates With Gut Metabolite Ptilosteroid A

**Figure 6:**
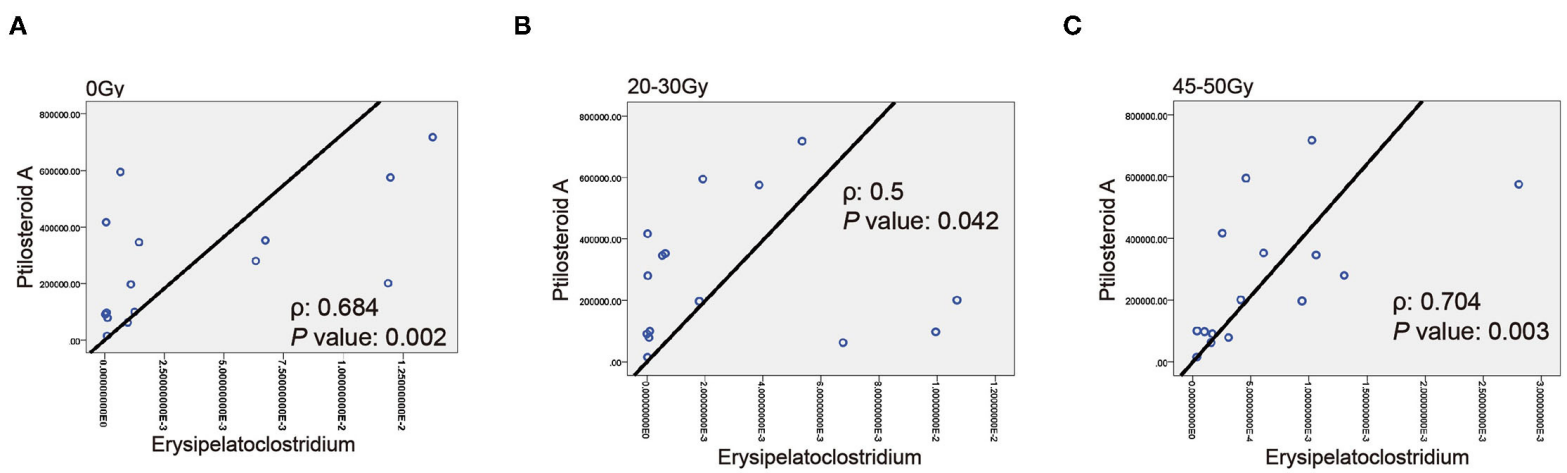
Associations between *Erysipelatoclostridium* and ptilosteroid A. **(A–C)** The correlation between gut *Erysipelatoclostridium* relative abundance and ptilosteroid A expression at all the three timepoints by Spearman's correlation analysis.

Next, the correlation between gut microbial *Erysipelatoclostridium* and gut metabolite ptilosteroid A was tested. We found a positive correlation between relative abundance of *Erysipelatoclostridium* and expression levels of ptilosteroid A at all the three timepoints (Figure 6), indicating that the gut metabolite ptilosteroid A could be mainly produced by gut microbiota *Erysipelatoclostridium*.

## Discussion

Based on the needs of manned return to the moon or Mars, the international aerospace powers focus on how to assess the space radiation risks. NASA summarizes the main research directions organized in recent years related to space radiation risks, which including radiation carcinogenicity, radiation-induced central nervous system damage, radiation-induced heart disease and other degenerative diseases, acute radiation syndrome immune system and radiation caused intestinal damage. After decades of research, a lot of evidence has been obtained about the effects of space radiation and health risks, but the underlying mechanism is still not fully understood, resulting in great uncertainty in space radiation risk assessment. The solution of these problems requires more space flight opportunities, more efficient simulation and monitoring technology of space radiation quality and biomarkers, and real-time on-orbit monitoring; technologies such as big data mining and biophysical modeling are needed to conduct space radiation, etc. Here, we wished to use a model of human radiation-induced intestinal injury to assess markers of space radiation induced damage to the digestive system.

Human intestinal microbiota refers to the microorganisms that live in the gastrointestinal tract, including bacteria, fungi, archaea, eukarya and protozoa, etc. These microorganisms inhabit the epithelial barrier surface of gastrointestinal tract, and exert both local and systemic effects (7–9). Increasing evidence has confirmed that gut microbiota orchestrates key physiological functions (10–12). Meanwhile, the out of balance of gut microbiota can lead to several local and systemic pathological processes, like obesity, cardiovascular diseases, autoimmune diseases, gout, inflammatory bowel disease, and several types of cancer (13–15). As for RIII, the role of gut microbiota in initially driving RIII has become increasingly evident (16, 17). In our study, we observed partly distinction of β-diversity but not α-diversity of intestinal microbiota between patients with grade 2 RIII and without. Then, we found that patients who developed grade 2 RIII had obviously enriched *Erysipelatoclostridium* after 45–50 Gy of RT. Univariate analysis showed that the relative abundance of *Erysipelatoclostridium* after 45–50 Gy of RT was related to the occurrence of grade 2 RIII. Furthermore, we developed a single gut microbial *Erysipelatoclostridium* model that exhibited high discriminative ability of RIII (AUC value of 0.85). Collectively, these available evidence indicated that gut microbiota including *Erysipelatoclostridium* may serve as potential bio-markers for RIII.

Since the 16S rRNA sequencing technology lacks the ability to tell transcriptional genes that each microorganism activates, gut microbiota can provide information on microbial structure rather than their functions (18). To overcome these deficiencies, gut metabonomics is an available option. In the gastrointestinal tract, these metabolites function as messengers, signal to distant organs in the body, and allow communication between local gut microbiota and distant organs (19, 20). For example, Mathewson et al. proved that gut microbiota-derived SCFA can mitigate graft vs. host disease (19). Recently, several studies have attempted to use gut metabonomics as biomarkers for RIII (21). Similarly, in our study, unique gut metabonomics signature of RIII was observed. Meanwhile, ptilosteroid A was more abundant in grade 2 RIII patients, especially after 45–50 Gy of RT. Univariate analysis showed that ptilosteroid A were related to the occurrence of grade 2 RIII. Further, we developed single variable model containing gut metabolite ptilosteroid A at three timepoints that exhibited high discriminative ability of RIII. Interestingly, better predictive ability can be obtained by combining ptilosteroid A of all the 3 timepoints. Ptilosteroid A was firstly isolated in the Solomon Islands in 2009 (22). Currently few articles about the function of ptilosteroid A has been reported, but the key role that lipids play in maintaining of gut epithelial integrity has been well-investigated. Therefore, gut metabolite ptilosteroid A may contribute to the etiology of RIII, and serve as potential biomarker for RIII.

Further, our study found a positive correlation between relative abundance of *Erysipelatoclostridium* and expression level of ptilosteroid A at all the three timepoints (0 Gy, after 20–30 and 45–50 Gy). As we mentioned above, ptilosteroid A belongs to the superclass of lipids and lipid-like molecules. Wang et al. reported that green tea leaf powder could improve lipid metabolism by modulating gut microbiota including *Erysipelatoclostridium* (23). Meanwhile, Luo et al. found that FuFang Zhenshu TiaoZhi could exert anti-aging effect *via* interference with lipid metabolism and by regulating gut microbiota including *Erysipelatoclostridium* (24). Therefore, gut metabolite ptilosteroid A could be mainly generated by gut microbiota *Erysipelatoclostridium*.

## Conclusion

In this prospective, observational clinical study, we identified alteration of intestinal microbiota, characterized by enrichment of *Erysipelatoclostridium*, elucidated disorder of gut metabolite, particularly in ptilosteroid A with RIII patients. We further identified *Erysipelatoclostridium* and ptilosteroid A could provide good diagnostic markers for grade 2 RIII. Next, multicenter, larger sample size trials are needed to confirm this.

## Data Availability Statement

The datasets presented in this study can be found in online repositories. The names of the repository/repositories and accession number(s) can be found at: https://www.ncbi.nlm.nih.gov/, PRJNA760986.

## Ethics Statement

The studies involving human participants were reviewed and approved by the Ethics Committee of The Second Affiliated Hospital of Soochow University. The patients/participants provided their written informed consent to participate in this study.

## Author Contributions

WM, YT, MW, and ML designed and supervised this study. SC, YY, YK, QG, YX, and PX recruited patients. YS, JQ, and RX were responsible for the follow-up. SC, LX, and YH conducted sample detection and statistical analysis. All authors contributed to the article and approved the submitted version.

## Funding

This work was supported by Young Talent Support Project of The Second Affiliated Hospital of Soochow University (XKTJ-RC202007) and Science Foundation of Jiangsu Health Commission (M2021081).

## Conflict of Interest

The authors declare that the research was conducted in the absence of any commercial or financial relationships that could be construed as a potential conflictof interest.

## Publisher's Note

All claims expressed in this article are solely those of the authors and do not necessarily represent those of their affiliated organizations, or those of the publisher, the editors and the reviewers. Any product that may be evaluated in this article, or claim that may be made by its manufacturer, is not guaranteed or endorsed by the publisher.
